# High performance coach cognition in the wild: using applied cognitive task analysis for practical insights–cognitive challenges and curriculum knowledge

**DOI:** 10.3389/fpsyg.2023.1154168

**Published:** 2023-06-29

**Authors:** Jamie Taylor, Michael Ashford, Matt Jefferson

**Affiliations:** ^1^School of Health and Human Performance, Faculty of Science and Health, Dublin City University, Dublin, Ireland; ^2^Insight SFI Centre for Data Analytics, Dublin City University, Dublin, Ireland; ^3^Grey Matters Performance Ltd., Stratford-upon-Avon, United Kingdom; ^4^Moray House School of Education and Sport, The University of Edinburgh, Edinburgh, United Kingdom; ^5^British Triathlon Federation, Loughborough, United Kingdom

**Keywords:** sport coaching, coach development, professional judgment and decision making, expertise, macrocognition, cognitive task analysis, elite coaching

## Abstract

Despite significant empirical work in the sport coaching domain, there remains a paucity of evidence to inform practice in high-performance sport coaching. As a result, there are gaps in our understanding regarding coaching expertise at different levels of athlete performance. A significantly underutilized approach in coaching research is Cognitive Task Analysis and it’s knowledge elicitation tools. Addressing these concerns, here we utilize applied Cognitive Task Analysis and a semi-structured interview protocol to elicit the cognitive challenges and use of knowledge by a group of *N* = 7 high-performance endurance sport coaches from a single national governing body. Analysis suggested prominent and ongoing challenges in day-to-day practice which, in turn require significant adaptive skill. In addition, results show how coaches used knowledge flexibly and conditionally to meet the demands of their role. A novel finding being the identification of the use of curriculum knowledge to mentally project the needs of athletes. The findings suggest opportunities for utilizing Cognitive Task Analysis to investigate the cognitive challenges of sport coaching and enhance coach development practice.

## 1. Introduction

As an applied domain, interest in the nature and function of sport coaching practice continues to grow in the literature (North, 2017). A key feature of this increased interest has been a desire to inform practice and generate implications for effective practice (Mallett and Lara-Bercial, 2016). This is especially important with increasing recognition of differences in environmental demands of specific coaching milieus. A result of which being the delineation of the coaching domains of children, recreational athletes and performance athletes (Lyle and Cushion, 2017). Similarly, an ongoing concern in the research literature has been the nature and identification of the type of athletes used in research samples and appropriate labeling of ‘elite’ athletes (Swann et al., 2015; McAuley et al., 2022). Despite sport coaching slowly emerging as a research domain, there has been limited recognition of these differences. As a consequence, there is a lack, there is a lack of empirical research generated in the domain of high performance (HP) coaching, defined as coaches working: “in contexts where emphasis is on preparation for and performance in competitive sport for achievement of performances that are, comparatively speaking, of the highest level” (Allen and Muir, 2020, p. 171).

Alongside these developments, there has been growing adoption of an expertise lens in sport coaching practice. The study of expertise has tended to concern itself with superior human performance and proficiency in complex domains spanning a range of communities of practice (Ward et al., 2019). This range of perspectives on expertise is no different in sport, with a variety of perspectives used to understand coaching expertise (e.g., Schempp et al., 2010; Turner et al., 2012). For instance, Nash et al. (2012) developed a criteria which served to operationalize and develop expertise in sport coaching. This work drew on insights from the Naturalistic Decision Making community of practice and Macrocognitive theory both empirically and conceptually (Abraham and Collins, 2011b; Harvey et al., 2015; Lyle and Muir, 2020; Taylor and Nash, 2023). Macrocognition explains a range of processes representing how experts think, including projection, flexecution, sensemaking, relearning and finding common ground. It is suggested that these processes underpin the management of uncertainty, detection of problems, decision making, co-ordination and management of attention (Hoffman et al., 2019). An advantage being the appreciative approach to understanding the cognition of experts in ecologically valid settings (Hutton, 2019) and understanding factors that enhance the development of expertise (Hoffman et al., 2014). Coach development has benefitted from this shift, with a move from a focus on competency to the acquisition of expertise (Collins et al., 2015; Cruickshank et al., 2020). One such development has been professional standards rooted in an expertise based approach and Professional Judgment and Decision Making (PJDM) in sport coaching practice (CIMSPA, 2019, 2021).

### 1.1. Professional judgment and decision making

PJDM is framed as an approach to practice that supports practitioner decision making in the complex social circumstances of coaching (Collins and Collins, 2015). Coaches will engage in processes of planning and re-planning based on changing situational demands, weighting of different agendas, drawing on a range of knowledge bases to engage in adaptive practice forms (Collins et al., 2022). Given the practical focus of PJDM, it has historically drawn on a range of theoretical perspectives on expert cognition. Using the pragmatic overlap between different theoretical stances to inform real world practice. For example, Abraham and Collins (2011a,b) drew on the work of Kahneman and Klein (2009) to recognize both the value of intuitive judgment and use of formalized procedure in goal directed decision making in coaching settings. A key feature being the role of more deliberative and more intuitive processes in expert coaching practice and the suggestion that deliberate thinking can enhance subsequent intuitive judgment (Klein, 2007). Thus, NDM and Macrocognitive theories have strongly influenced the growth of PJDM as an approach to professional practice (see Table 1).

**Table 1:** Definitions of relevant concepts.

Concept	Definition
Adaptive Skill	“Timely changes in understanding, plans, goals, and methods in response to either an altered situation or updated assessment of the ability to meet new demands, that permit successful efforts to achieve intent or successful efforts to realize alternative statements of intent that are not inconsistent with the initial statement but more likely to achieve beneficial results under changed circumstances” (Ward et al., 2018, p. 42)
Expertise	“The characteristics, skills and knowledge that distinguish experts from novices” (Ericsson, 2018, pp. 3–4)
Macrocognition	The dynamic application of thinking to evolving events including decision making, situation awareness, planning, problem detection, option generation, mental simulation, attention management and uncertainty management (Hutton, 2019)
Naturalistic Decision Making	A theoretical lens and applied methods to study expert decision making (Klein, 2008)
Professional Judgment and Decision Making	An approach to applied practice promoting informed decision making based on contextual needs (Martindale and Collins, 2005, 2007)

### 1.2. Adaptability

Taking account of the fundamental underpinning of Macrocognition as being the “adaptation of cognition to complexity” (Hoffman et al., 2009b, p. 87), adaptability sits at the heart of Macrocognitive functions. Adaptability has increasingly become a feature of the coaching literature, though to this point empirical investigation has tended to focus on adventure sport coaching, rather than more traditional sport contexts (e.g., Mees et al., 2020). This is perhaps a result of the environmental context of adventure sport, which Collins and Collins (2022) suggest is information-rich and hyperdynamic (Prinet et al., 2016). Coaches are therefore faced with ill-structured problems, requiring assessment and rapid decision-making under time-constraints (Collins and Collins, 2017). To theoretically frame this need for adaptability, authors have tended to draw on the work of Hatano and Inagaki (1986) and the notion of routine and adaptive expertise. Routine expertise captures the practice of an individual who has developed a set of competencies which allow them to satisfy specific tasks (Bohle Carbonell and Dailey-Hebert, 2021). However, reliance on such routines when dynamics of a situation changes, may result in a breakdown in performance, or more specifically for coaches, an inability to effectively notice and support the needs of an athlete (*cf*. Schwartz et al., 2005). More recent literature has questioned the concept of the routine expert, making a coherent argument that adaptive skill is the “conditio *sine qua non*” of expertise (Ward et al., 2018, p. 35). Thus, there is a need to understand adaptive skill as it relates to the HP sport coach.

### 1.3. Coaching knowledge

Building on an understanding of the coaches’ ability to use adaptive skill, previous literature has suggested the need for the use of knowledge to take action against what is perceived in the environment (Abraham and Collins, 2015). Knowledge and its use has long been an area of interest among scholars in coaching (North, 2017). Different bodies of knowledge have been identified as of importance including knowledge of the sport (technically, tactically), of the person (including ‘ologies–e.g., psychology, physiology) and pedagogy (the science and practice of coaching) (Abraham et al., 2009). In addition to identifying bodies of knowledge, a variety of typologies of knowledge have been developed. A predominant framing in the coaching literature being declarative (of concepts and principles); procedural (of skills and strategies) and tacit, acquired through experience (Nash and Collins, 2006). In the broader literature, these labels have received some critique, with alternatives such as conceptual, experiential and conjectural being proposed (e.g., Hoffman et al., 2014). Specifically in sport coaching, the absence of declarative or conceptual knowledge appears to be a barrier to changing practice (Stodter and Cushion, 2019). Kirschner (2009), p. 147 captures this simply, as: “what experts already know determines what they see and how they see it.” Regardless of framing, it has been widely suggested that flexible practice is enabled by the recognition of salient patterns, developed through the acquisition of conceptual knowledge over time.

Literature has also drawn attention to the practical necessity for knowledge to be considered in contextual terms and the importance of conditional knowledge (Collins et al., 2012a). Conditional knowledge being the knowledge of when and why forms of knowledge are useful (Schunk, 2012). In practice, this has suggested the need for practitioners to apply a level of criticality in their acquisition of knowledge (Stoszkowski et al., 2020) and for different knowledge bases to be applied flexibly (Grecic and Collins, 2013; Crowther et al., 2018). This conditionality and the need to use knowledge flexibly and contextually across different areas is a critical element of expertise (Hoffman et al., 2014). Extending these assertions, the Data-Frame model of sensemaking proposes an abductive process whereby frames are used as top-down explanatory structures to account for bottom-up data (Klein et al., 2006a,b). That, in essence, knowledge and pre-existing framing acts as the means by which we understand and perceive data (Klein et al., 2006b). This suggests that experts and novices do not differ in terms of cognitive processes, but instead by content and knowledge bases used in the sensemaking process (Sieck et al., 2007). Thus, effective practice depends on knowledge structures held by the practitioner (Hoffman and Militello, 2009).

Importantly, where expertise models have been used to inform coaching practice, knowledge is considered a part of what underpins practice, rather than the full picture. As an example, Nash et al. (2012) also identified the need for: the utilization of perceptual skills, mental models, a sense of typicality and routines; the ability to work independently producing novel and innovative solutions, effective use of reflection and lifelong learning, understanding of own strengths and weaknesses and managing complex planning processes. In short, representing the range of Macrocognitive functions identified elsewhere in the literature (Klein et al., 2003). More recent literature has continued this trend with Collins and Collins (2022) drawing on the work of Adams et al. (1995) to explain adaptive skill. They suggest the need for a synergy between the use of knowledge, available information and exploration of the environment, to tackle the cognitive demands of coaching. This therefore suggests the opportunity for an exploration of expert sport coach cognition and the role of knowledge.

### 1.4. Investigating sport coach cognition

The validity of methods used to investigate the cognition of coaches has been a matter of debate in the literature (Lyle and Vergeer, 2013). A variety of methods have been used, for example: stimulated recall (Harvey et al., 2015), scenario based approaches (Morgan et al., 2013), think aloud (Swettenham and Whitehead, 2022) and mixed methods (Ashford et al., 2022). An alternative series of methods, adopted across expertise studies are the family of Cognitive Task Analysis (CTA) tools, which have been developed and flexibly deployed across a range of professional settings (Graham et al., 2022). CTA is used as a means of eliciting expert cognition and knowledge in both specific and more global situations (Hoffman et al., 1998). CTA tools allow for the investigator to understand the cognitions of the person performing the task (Klein and Militello, 2001) and generate data against practitioner knowledge and reasoning processes (Hoffman and Militello, 2009). A specific approach to CTA is Applied Cognitive Task Analysis (ACTA) which provides the opportunity for general domain application and flexibility (Hogenboom et al., 2021). Further, allowing for the explorations “of and in professional knowledge management practice” (Gore, 2013, p. 203).

ACTA is a logical progression of knowledge elicitation and representation methods which aim to capture difficult judgments, attentional demands, critical cues and strategies used by professionals (Gore et al., 2018). Importantly, given the scope of the investigation, CTA tools can offer the flexibility to discover a broader understanding of the demands and complexities of a domain, along with general knowledge and skills. CTA can also be employed to offer an iterative and detailed analysis of specific tasks or roles in a discipline (Hoffman et al., 2009a). Despite Nash et al. (2012) suggesting that ACTA could be used as a means of understanding “what (coaches) know and what they can do with that knowledge” (p. 8), significant use of Macrocognitive modeling in sport (Ashford et al., 2021; Bossard et al., 2022), and the recommended use of CTA methods (Lyle and Vergeer, 2013), there has been limited use in sport coaching. To this point, ACTA’s use has been limited to understanding the management of cognitive load of adventure sport coaches (Collins and Collins, 2021), the processes of coach developers (Abraham, 2016), the decision making processes of early career (Downes and Collins, 2021b) and high level (Downes and Collins, 2021a) strength and conditioning coaches. Set alongside the complex nature of coaching practice (Cushion, 2007), this is perhaps surprising given ACTA’s context sensitivity and potential for ecological validity (Gore and McAndrew, 2013). As a result, the field is missing the use of a range of robust knowledge elicitation tools (e.g., Crandall et al., 2006), many of which subsequently being used to inform the design of training (e.g., Patterson et al., 2016). Consequently, as suggested in the study of athlete cognition (Richards and Collins, 2020), there is an opportunity for ACTA to enhance our understanding of the cognitive challenge of HP coaching and contribute to the evidence base in coach development practices.

Forming part of a broader project to inform coach development in a successful Olympic and Paralympic sport, this study has two specific aims: (a) elicit the cognitive demands faced by expert coaches at different levels of athlete performance and how coaches manage these demands and: (b) explore the coaches’ use of knowledge to tackle the demands they face. In both instances, the research questions were shaped by the practical needs of the third author as an experienced coach developer in a National Governing Body.

## 2. Method

### 2.1. Research philosophy

The exploration of pragmatic knowledge sits as a center piece of this piece of work; logically therefore, a pragmatic research philosophy has underpinned its execution (Cruickshank and Collins, 2017). Advocates of pragmatism have suggested that knowledge, generated through research, should be conducted based on the impact and difference it makes in practice (Giacobbi et al., 2005). Furthermore, pragmatism places the research question at the heart of any methodological decisions, rather than adherence to methodologies advocated by authors from specific ontological and epistemological perspectives (*cf*. North, 2017).

### 2.2. Participants

Based on the aims of the investigation it was necessary to recruit coaches with a track record of working with both TD and HP athletes, the latter meeting the definition offered by Allen and Muir (2020) and thus currently coaching athletes at the Olympic and Paralympic level. Recognizing that the coaching of elite athletes does not confer a level of expertise on the coach (e.g., Blackett et al., 2017), in addition the guidelines developed by Nash et al. (2012) were used as a sampling criteria. As a result, coaches were nominated by a single national governing body (NGB) based on peer recognition of (a) a track record of innovative coaching, as recognized by the HP coaching community; (b) attitude toward reflective practice and learning; (c) management of complex planning processes; (d) a track record of developing athletes to the elite level and (e) success at the Olympic or Paralympic level. In turn, matching the criteria suggested by Crispen and Hoffman (2016) of career, sociometric and performance analysis. Thus, NGB recommendations led to a group of *N* = 7 coaches being contacted, provided with information regarding the study and all subsequently provided informed consent to agree to a series of three interviews. The sample contained coaches with experience of coaching senior international athletes to medal winning performances and extensive experience of coaching athletes at talent development level. The sample also represented coaches from both Olympic and Paralympic disciplines. A particular advantage conferred by the context were that coaches had actively engaged in the coaching of athletes at different ages and stages, across boundary markers that may distinguish between TD and HP coaching contexts (Lyle and Cushion, 2017).

Although ACTA guidance typically recommends the use of 3–5 subject matter experts (Militello and Hutton, 1998), the number of participants recruited for the study was higher (*N* = 7, *N* = 1^female^ and 6^male^). The reasoning for the slightly larger sample was both the availability of expertise and breadth of investigation. Based on the necessity to protect participant anonymity, no further demographic information is presented, and data is unavailable on request.

### 2.3. Procedure

Following ethical clearance from Dublin City University (REC/2022/171) a three stage ACTA was completed in full, but given the breadth of focus for data collection and the flexibility of the tool (Minotra and Feigh, 2017), an adaption to the protocol was made with the addition of a semi-structured interview following the task diagram. The three stages were conducted across three interviews by the first and second authors. Due to the depth of investigation, data was collected over a period of weeks, with at least 5 days between episodes of data collection. Interviews were conducted online *via* video conferencing software, during a phase of the year when the majority of athletes that the coaches worked with would be considered as being in a ‘competition phase’.

#### 2.3.1. Trustworthiness

As the research utilized a blend of qualitative approaches, several guidelines were deployed to enhance trustworthiness. Drawing on recommendations in qualitative research a broad approach to member reflection was deployed in multiple formats (Smith and McGannon, 2018). Facilitated by the third author, this saw multiple rounds of presentation and reflective sessions to coaches engaged in the project and other members of staff who witness the day to day working practice of the engaged coaches. Participants in these groups were asked about the fairness and appropriateness of the cognitive demands and the themes generated by the researchers using TA. In all instances feedback was positive and perceived to be representative of the day-to-day cognitive challenges faced by coaches. Reflections from coaches also drew attention to the demands and the interpersonal demands imposed by the need for athletes to consider the coach professionally competent. In addition, reflections from focus groups further emphasized the meaning and importance of mentally projecting the needs of athletes over time.

There are limited guidelines that have sought to establish quality in CTA research. As such, the research was guided by the suggestions of Roth et al. (2014) in seeking to offer ‘quality’ by offering an appropriate level of rigor and clear application of findings for the betterment of a domain. CTA methods, while open to critique on the basis of reliability and falsification, are highly appropriate to the pragmatic research philosophy adopted. Consequently, we ask the reader to judge quality and trustworthiness by the following markers: firstly, by outlining the quality and quantity of SMEs that were interviewed in the project. Secondly, the quality of data generated by ACTA methods depends to an extent on the skill of the interviewer (Hutton and Militello, 1997). The first two authors had undertaken training in CTA methods (CTA Institute), had previous experience with the use of ACTA and were experienced qualitative researchers. In addition, given the intellectual challenge posed by analysis of intuitive processes captured through more deliberative reflection (Kahneman and Klein, 2009), there is a need for the researcher to engage in careful interpretation and have significant previous experience (Gore et al., 2018). This interpretation was supported by the research team’s experience as coach developers. Similarly, in the first author’s publication record, applying deliberative and intuitive approaches to coaching practice (Taylor et al., 2023). Thirdly, planning for the CTA was evaluated *a priori* by an experienced CTA researcher outside of the research team as a critical friend (Smith and McGannon, 2018). Finally, another marker of quality is the extent to which the CTA Offer insights that deepen our understanding of a particular domain. We leave this judgment to the reader.

#### 2.3.2. Task diagram

**Figure 1:**
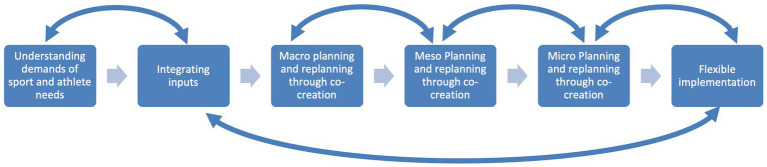
Coaching process task diagram.

The Task Diagram step is typically used to conduct an initial screening of areas of practice might pose the most difficult cognitive challenges (Crandall et al., 2006). A two-step process was used, firstly using the typical Task Diagram approach, firstly stating that the ‘task of interest’ was understanding a general overview a coach’s general approach to the development of their athletes. Following existing guidance, probes were then used to understand the steps taken by coaches, for example: “at the most basic and simple level, can you break down your coaching process into between three and six steps.” The second half of the interview employed a semi-structured interview guide, which was used to understand the impacts of coaching on successful (Olympic or Paralympic champion) athletes, against those who appeared to be high potential but fell away (*cf*. Taylor and Collins, 2019). This began by using the task diagram as a scaffold, asking them what key features of each stage enabled or disenabled athlete development and on a second sweep, what effective and ineffective coaching environments offered athletes. The purpose being to check for and probe the perceived critical elements of their practice. In several instances, this second sweep led to coaches suggesting subtle variations on the steps adopted. Following initial explanations, the task diagram and semi structured interview, lasted a mean 107 min (SD = 6.36). Due to the richness of data collected, an exemplar rather than summary of coaching process is presented (see Figure 1). Importantly, this highly simplified representation of the processes that coaches discussed was not linear or sequential. We aim to demonstrate this non-linearity by presenting linkage between steps.

#### 2.3.3. Knowledge audit

The second interview utilized a knowledge audit with interviews lasting a mean 118 min (SD = 27.7). The purpose of the knowledge audit is to identify elements of the coaching process requiring judgment and decision making, accordingly, identifying the knowledge bases that underpin this expertise. The knowledge audit therefore offers a general survey of the cognitive challenges associated with a domain, along with the related knowledge, cues and strategies (Militello and Anders, 2019). Utilizing the output from the expanded task diagram interview, the knowledge audit asked coaches to reflect at three levels of analysis (macro, meso and micro). Probes were designed based on commonalities in expertise across domains and were designed to identify cues, strategies and cognitive difficulties (Militello and Hutton, 1998). They were also adapted to meet contextual conditions and jargon of the coaching domain. Examples of each are presented in Table 2, adapted from Militello and Hutton (1998). Following each probe, coaches were asked “How would you know this? What cues and strategies are you relying on?,” and: “how would this be difficult for a less-experienced coach?”

**Table 2:** Knowledge audit probes.

Probe	Description
1	Past and future: “Can you remember entering a coaching situation when you knew how things got there and where they were headed?”
2	Big Picture: “What are the big picture targets you are aiming for?” “Can you explain how you go about this stage of your coaching process?” “What are the main elements you need to track as things progress?” “How did you decide on a focus for each session/athlete?”
3	Noticing: “In this process, can you remember any element of a situation popping out at you that others did not notice?”
4	Tricks of the trade: “Are there any ways of working that you have found to be more effective or efficient?” “Who else is involved?”
5	Improvising/opportunities: “Can you think of a time when you have improvised, or noticed an opportunity to do it better?”
6	Self-monitoring: “Can you remember a time that you needed to change the way you were coaching to get a good outcome?” “How did you make adjustments?”
7	Anomalies: “Can you remember a time that you knew something was amiss?”
8	Information Difficulties: “Have you ever had a time that data or multidisciplinary input pointed in one direction, but your judgment suggested something else?”

#### 2.3.4. Simulation interview

The third stage of data collection utilized the simulation interview to elicit a view of coaches’ problem-solving processes in context. Building on the first two stages, we aimed to develop a deeper understanding of the complex issues encountered by this group of coaches. Instead of simulating a particular coaching task, we requested coaches to reflect on three distinct and demanding coaching episodes, each involving athletes at different levels. Coaches were then asked to list all the major events along the timeline of a specific incident, these were identified as key judgments or decision points. As these were listed, they were recorded and listed in the left-hand column by the researcher. Subsequent columns were used to record actions, probed by: “as the coach, what actions did you take at this point?”; situation assessment: “what did you think was going on at this stage?,” critical cues “what information led you to believe this?” and finally, potential errors: “what errors would an inexperienced coach make here?.” This stage of the ACTA lasted a mean 99 min (SD = 8.88).

### 2.4. Data analysis

#### 2.4.1. Cognitive demands table

The final stage of the ACTA, the Cognitive Demands Table provides a framework for evidence informed implications (Hutton and Militello, 1997). These enable the presentation of more tacit features of expertise in the form of key cues and strategies focusing on the outcomes of analysis that are pertinent to problem solving and decision making (Gore and McAndrew, 2009). Following each manuscript being transcribed verbatim, the established process of analysis used by McAndrew and Gore (2013) was followed and each element of expertise considered sequentially. Theme generation was conducted by synthesizing common participant responses to individual probes based on their salience and frequency.

#### 2.4.2. Reflexive thematic analysis

Due to the exploratory nature of the second research aim, a Reflexive Thematic Analysis approach was used to explore how coaches used knowledge (Braun and Clark, 2022). Aligned to the pragmatic research philosophy, the researcher’s backgrounds as experienced coaches, coach developers and researchers was considered a strength and a resource to inform TA (Braun and Clarke, 2020). As is typical when using TA, analysis took place over six-stages (Braun and Clarke, 2006). In this instance however, data analysis was integrated with the ACTA. Therefore, the first step of data familiarization involved reading and re-reading transcripts and the compilation of the cognitive demands table. The second phase led to code generation related to the coaches’ use of knowledge over multiple sweeps of the data. Codes generated were both semantic (e.g., ‘multiple knowledge bases’) and latent (e.g., ‘experiential understanding’). At the third stage, initial themes and sub-themes were generated to represent clusters of shared meaning (e.g., ‘integration of knowledge bases’). At the fourth stage, initially the first author reviewed the initial theme generation against the broader data set, checking for meaning and coherence. This led to the clustering of two main themes given shared meaning. The fourth stage was continued by the second author who performed a similar sweep to check for coherence with no additional changes. At the fifth stage themes were defined and named based on these patterns of shared meaning. The sixth and final phase was the write up of themes which are presented in the Findings section.

## 3. Findings

The findings are presented in two overarching sections to address respective research aims. The first section addresses the aim of synthesizing the cognitive demands faced by coaches as the final stage of the ACTA process. Each elicited cognitive demand is outlined using thick descriptive quotations from participants (Tracy, 2019). The second section presents generated themes exploring coaches’ use of knowledge to manage cognitive demands. Quotations are used throughout to orient the reader, however, any quotations that may have compromised the anonymity, competitive advantage of coaches or athletes have been excluded or redacted. Quotations were selected based on the criteria of authenticity, that they illustrate the nature of the phenomena, are appropriately succinct and are representative of the dataset (Lingard, 2019).

### 3.1. Cognitive demands of coaching

Each cognitive challenge represents an ongoing difficulty faced by the coaches in their day-to-day practice (represented in Table 3). The most prominent cognitive demands for coaches were: making sense of individual context, planning for priorities, stress management for the athlete, when to push or pull, managing the coach-athlete relationship, orchestrating inputs to the athlete.

**Table 3:** Cognitive demands table.

Cognitive demand	Why difficult?	Cues	Strategies	Potential errors
Making sense of individual context	“Coaching is about a million different things” Conditional application of knowledge Fitting individual plans with the group/working within the typical season plan Understanding the sport Depth of demands Application of physiological demands Breadth of knowledge “70% of the specialist”	Take account of whole context Tracking/monitoring progress–recognizing non-linearity of progression How the athlete ‘looks’ Taking account of evidence from training and ultimate performance	Clarity of intention and subsequent appropriate reflection “Plan in pencil, not pen” – flexibility of planning Consistency of staff Consistency of coaching approach with individuals Seek support outside areas of expertise and use of practitioner group Helping athlete understand why (role clarity) Supporting development of independence/understanding for athletes Linking the micro to the macro	Not knowing the athlete Not utilizing views of others Making assumptions and rushing the planning process Limited attention to the impact of small decisions Limited knowledge base
Planning for contextual priorities	Picking appropriate interventions Maintain focus on what is important, i.e., understanding minimum effective dose Checking for progress Balancing what is needed to perform at the top vs. what might stop athletes progressing	Seeing the links between specialisms/knowledge bases Monitoring athlete response to stimulus Comparing current status with desired future	Knowledge across domains to support interventions Integration of support Knowledge of calendar, macro/meso planning Consistent performance testing Mental projection Drawing on previous experience of athletes	Not having experienced previous athlete progression Lacking curriculum knowledge Jumping to solutions without taking time to consider problems Assuming linear athlete development
Stress Management for the athlete	Highly committed athletes Potential for multiple training environments Complex interplay with broader life Growth/maturation for younger athletes Competing interests for the athlete Large groups	Athlete feedback Intuiting athlete status Progression of training load Individual responses How the athlete ‘looks and moves’ Checking for boredom Wider life of the athlete Consistency of menstrual cycle Dexa scanning and bone health	Balancing volume with robustness training Coherence of coaching Managing athlete role clarity Help athlete understand why Profiling (physiology) Promoting recovery Incremental novelty in session planning	Not managing athlete expectations Not putting athlete health before athlete wants Not adjusting plan Not seeing the rest of an athlete’s life Not recognizing a lack of robustness Seeking rapid progression of performance
When to push or pull?	Highly committed athletes Preventing a level of comfort and ensuring accountability Time needed to know the athlete The norms of the sport Balancing wants/needs of the athlete Different needs within a training group	Understanding bandwidth for compromise Norms for each individual Adapting based on athlete status Fatigue of athlete Checking progress against goals Level of athlete independence Individual athlete response	Understand background of the athlete Meet the athlete where they are Prioritize key sessions Knowing when to give them enough of what they are good at Know when to reassure and when to challenge Prioritizing health above athlete wants	Not individualizing Overly dictatorial or overly accommodating Lack of previous experience Leaving it to athlete preference when wellbeing is at stake Lack of 360 communication
Managing the coach-athlete relationship	Caring while maintaining professional distance Balance conveying competence/openness Potential to break trust Providing appropriate support	Sensing what is going on for them/ noticing change Hearing their perspective Knowing self Having a shared language	Maintain role clarity for athlete Cultivate perceived competence with the athlete Invest time with each athlete Cultivate openness Managing interpersonal approach with athlete (body language etc) Maintain open communication with others around the athlete	Unbalanced approach (all athlete or coach) Not taking athlete ‘literacy’ into account Being overly friendly and not performance focused Not admitting errors
Orchestrating inputs to the athlete	Navigating micro-politics Individualizing v personalizing Balance of individual vs. group training Stress management Creating appropriate competition Managing conflict	Awareness of number of inputs to the athlete Awareness of strongest influences on the athlete Awareness of group dynamics	Promote adaptive role models Generate competition Build role clarity in practitioner support group	Allowing too much external influence Not knowing what was going on in the group Getting the balance right between group training and individual needs

#### 3.1.1. Making sense of individual context

The first synthesized cognitive demand was the challenge of being able to make sense of the individual context of the athlete. This related to the challenge of the coach being able to weigh up the unique and individual needs of athletes and also that of their context. This challenge was often driven by the complex interaction of factors impacting the work of coach and athlete. Coaches described this challenge as being a matter of

There is just so much to take account of… you need to make sense of things as they happen, you need to continuously update your mental model of what they [the athlete] need and the changing demands of the sport (C4)

Cues and strategies used to manage the challenge were varied but related to an observation of how the athlete looked and responded during interactions. Thus, coaches deliberately adopted strategies that enabled a level of consistency in their own approach. This was seen to support the use of incoming data regarding the athlete, as it would enable observation of the athlete’s response to training. This was partly managed as a function of the time that athletes and coaches spent together, where coaches felt that by deliberately investing time in getting to know the athlete and their responses, they were better able to frame their observations.

It was also supported by a heuristic notion of “planning in pencil, not pen,” a recognition that they could not become too fixated on a particular course of action and instead needed to engage in the rapid ‘sizing up’ of situations.

It is a mistake to think you will get linear athlete progression, things change, and anyone that’s involved with athletes knows that it’s never linear…you need to have a longer term plan and know where your athlete is placed within it, but to have something super concrete, it’s just a waste of time, the context always changes, you can’t be locked into something that you have no control of (C2)

Another prominent strategy used was the use of other coaches and specialist multi-disciplinary staff to audit initial observations and support sensemaking. In addition, actively developing the ability of the athlete to provide appropriate feedback on their state and giving them role clarity.

It was suggested that less experienced coaches would make conclusions about athlete and contextual needs too quickly:

There is a lot that goes into coaching athletes at this level, a less experienced coach would rush things or make assumptions…it is recognising all the different factors that are going on and being able to work with them. That’s the key, it’s the ability to read the situation (C3)

There was a view that these errors were grounded in an overconfidence in their individual coaching frames and not on the contribution that other practitioners or the athlete themselves could offer.

#### 3.1.2. Planning for contextual priorities

The second cognitive demand concerned the ability of the coach to engage in deliberate longer-term planning. This involved a high demand on the coach being able to identify different trade-offs and weigh up priorities as needed for the individual athlete. This meant that there appeared to be no single means of operation for the coach, they were consistently dealing with cognitively challenging trade-offs:

Everything is a trade-off, it all depends. So if you decided that the way to go faster was developing XXX, the way to do that would probably be to increase XXX, but that negatively impacts XXX. Equally to better your performance at XXX, you need to be more XXX. So therefore, you need to reduce XXX and be better at XXX. The consequence is that it negatively impacts XXX (C3)

A core feature of this deliberate thinking was the need to consider the specific priorities for athletes. This was a particular concern for athletes who were considered world class or near Olympic/Paralympic champion performers where the margins of error were small and the ability to make change was limited:

One of the critical determinants of a coach at world level, is being able to figure out what priority you have and go: ‘this is what we’re working on’. It is the ability to go: ‘here’s a box of 50 things, this is the one that’s going to make the performance impact, this is the one that’s going to make the difference’ (C1)

The process of prioritization also involved coaches mentally projecting the consequences of subtle changes in situational demands and how they might respond to these changes:

I’m constantly weighing everything up and there is a lot to weigh up. It is always changing so I am probably in permanent micro planning mode. I get it when I think about it, but it feels very instinctive (C1)

In essence, coaches were always balancing complex planning and prioritization processes. These trade-offs were faced on a day-to-day basis and imposed significant cognitive load.

#### 3.1.3. Stress management for the athlete

Given the nature of both the HP milieu and the aerobic demands of the sport, it is perhaps unsurprising that the management of overall stress was an ongoing and prominent challenge for coaches. Thus, coaches engaged in a variety of mental projection in relation to the desirable consequences of stress and the athlete’s ability to adapt to a variety of imposed stressors.

I’ve never found an athlete who’s that capable of being totally objective about how they are feeling. You need to help guide them with those decisions over a long period of time…you either get it right or get it wrong by overstressing and not recovering enough. What follows is overtraining, injury, or long-term illness. On the flip side, you don’t stress enough, and you don’t get progression (C2)

The challenge of managing and imposing appropriate stress led to coaches needing to perform a variety of workarounds. This was in part driven by the perception of limited validity in the markers that coaches could use to guide their decisions and further challenged by the inter-individual variability in response to similar training. Coaches reported frequently relying on the perceptions of their athletes, which, in itself, presented a cognitive challenge:

I’ve found that some athletes can ramp up pretty quickly to do 25 to 30 hours [of training per week] and be fine, wanting more and be able to cope. I’ve also found athletes who just can’t do that, over 20 hours a week they struggle. There is a little bit of trial and error…obviously making a plan, the simple stuff of not increasing too much. The real trick is balancing the XXX [training volume] because we often see that if you ramp that up too quick, we’ve got a lot of lower limb injuries. It is still very individual though (C7)

This was made even more cognitively demanding by the holism of the stress response and recognition that the athlete’s ability to adapt depended on a constellation of other factors outside of the training environment. Thus, coaches paid attention to the types of cues recognized as being important, including the athlete’s body language, discussions about their wider life and the integration of a range of information about them. Coaches also employed the deliberate strategy of making subtle, rather than significant changes to training as a means of testing response:

I will make daily adjustments to the overall plan based on how the athlete responds, not just to training but everything else that is going on, they might only be little ones, but it will never play out exactly as you thought it would (C3)

As a result, coaches perceived the need to have to make continuous, often intuitive judgments to adjust training, both based on a sense of typicality for the athlete and longer-term planning.

#### 3.1.4. When to push or pull?

Linked to the provision of appropriate stress for the athlete was also a view that coaches needed to make decisions regarding their pedagogic stance with the athlete. Thus, making appropriate decisions about the extent to which they engaged in more ‘push’ like approaches in their coaching where they held the athlete accountable and encouraged higher levels of effort and attention. Or, in contrast, adopt a more ‘pull’ based approach, where there was a need to reduce the athlete’s effort and encourage more recovery. This was also the case for coaches deciding on the role they played pedagogically and the stance they adopted toward their athletes with different people requiring different approaches:

It’s almost a spectrum between the role you play as a coach that ranges almost an instructor, to collaborator, advisor, consultant – something like that. I would know, somewhere on that spectrum is where each of the athletes want me to sit. I try hard to be more of an educator and not just instruct them but with some athletes, it’s difficult, that’s what they want (C4)

The particular challenge in this instance were the social dynamics and being able to offer a range of different approaches to those in a coaching group, depending on the individual needs of the athlete.

The differences between athletes are acute, so coaching is signficantly different between people, what they need and want from coaches and people are different. Then for me understanding, why they might need to back off for a session, but they think ‘I’m fine’, or why they need to push a session when they’re not wanting to push, or why this might have changed. If you’re changing things, just having a really clear rationale that again, the athlete not just understands but believes it is in their best interest (C6)

This was also partly in recognition of the preferences of each individual athlete, where at this level of performance athletes were perceived to need to engage with activities that involved areas of weakness or were physically and mentally demanding:

Hate is probably too strong a word but there’ll be things that athletes dislike doing. The best way to manage that stuff is that the athlete knows why they’re doing what they’re doing. Even if it’s at a regurgitation level, even if they go: ‘oh, I didn’t like but it’s to toughen me up in open water so that when I get to the first XXX, I’ll feel better’… Basically as a coach I need to span the balance between pushing and pulling. It is something I constantly wrestle with; how dictatorial should you be with somebody? With XXX she needed and wanted me to be more dictatorial. I wasn’t comfortable with being that dictatorial, maybe I got it wrong (C1)

The challenge of adjusting a particular coaching approach was also challenging based on individual difference, not only in terms of preferences, but also what they need from a coach based on objectives and ambitions:

#### 3.1.5. Managing the coach-athlete relationship

In addition to the challenge of understanding the appropriate orientation for coaches to approach their practice, coaches also reflected on the challenge of managing their relationship with athletes. This was driven by the anticipation of future states conferred by the nature of the coach-athlete relationship. This was challenged, both by the strength of culture in the sport as one where the athlete utilizes the coach, rather than the coach being the dominant figure and by the frequency of contact between athlete and coach. Thus, coaches discussed the challenge of managing and maintaining appropriate professional distance, to prevent negative consequences of being overly close and preventing the athlete becoming dependent on the coach:

You absolutely cannot be mates, you are of course friendly, but there has to be a professional distance and separation between being friends and being a coach and an athlete. If you’re too close, you can’t have the ability to step back and have the conversation that you need to have. They rely on you to be a coach, not a mate (C1)

Coaches utilized a range of strategies to manage this cognitive challenge, including ongoing contracting to maintain role clarity and subsequent investment of time within these boundaries. This role clarity was deemed particularly important during periods of disagreement:

You might not always agree with things that the athlete thinks but you navigate through it. It is mostly through having built developed trust with those individuals…You’re never fully in charge, you can only help and advice. They’ve got to want involvement from me, that’s based on believing I can help them get better (C5)

The nature of the interpersonal challenge was perceived to be strongest when working with the highest performers: “with elite athletes, they are more opinionated, they believe in what they are doing” (C2). Coaches managed this challenge by cultivating and generating a perception of competence:

One of the most difficult things is demonstrating the right level of competence to the athlete so that they have confidence in what you see and what you’re talking about. What they believe has a massive impact on the adaptation (C6)

By demonstrating higher levels of competence, this was perceived to elevate trust and enable greater experimentation and openness at other stages. However, this was also seen as a delicate balance. Coaches suggested that while athletes may lose confidence in them based on a lack of perceived competence. The loss of trust because of a failure to admit errors and offer an appropriate level of openness was perceived to be a typical error for the less experienced coach.

#### 3.1.6. Orchestrating inputs to the athlete

The final cognitive challenge was the active orchestration of the wider milieu and other inputs to the athlete. This complex micro-politicking required the coach to monitor and engage with a variety of social feedback loops. It required the identification of priorities and navigating the complexity of social systems. As an example, coaches often sought to utilize more senior athletes as a means of role modeling and tackling perceived issues relating to the culture of a group:

It is a lot of ongoing individual conversations and it is very difficult to get it right, but we message through the older athletes. We’ve often found that if we get it right, older athletes have bought into and educate younger athletes. I often find that the athlete says it is way more powerful than when a coach says it. You know, with XXX she’s turning around to an 18 year old going: ‘why are you doing that?’ They’ll stop and listen immediately. Whereas if a coach says it, they might potentially ignore it (C7)

Given the breadth of experience in the sample, coaches were in a position to discuss the differences between HP and younger athletes. At developmental stages, this complexity often came as a result of the variety of stakeholders around the athlete, such as parents and coaches. With elite athletes, coaches were responsible for the coordination of multidisciplinary teams with a multitude of different support services that input to the athlete.

The coach…is probably responsible for initiating the support from staff. The role is a big team player, because they have to work with your head of [sports science and medicine] services and each domain practitioner. The problem is for the coach to have the ability to systematically bring all those people together and get it right for the athlete. Sounds really simple, but it’s really difficult …keeping everyone up to speed and aware of priorities and keeping people coherent (C6)

Doing this effectively required skillful social orchestration on the behalf of the coach, understanding and navigating the micro-politics of the coaching environment. Less experienced coaches were seen to make errors in this regard by not taking the time to ask athletes about the broader group and take the time to understand social dynamics. Key cues to manage this demand included ongoing and frequent conversations with athletes and those around them to check for coherence.

### 3.2. Coaching knowledge

The second research aim was to explore coaches’ use of knowledge in managing cognitive demands (see Table 4). Generated themes are presented below with the themes: ‘breadth and depth of knowledge’ and ‘flexible and contextualized use of knowledge’, each sub-theme is presented in italics. All themes are presented with a conceptualization of meaning (Braun and Clarke, 2023).

**Table 4:** Coaches’ use of knowledge.

Theme	Sub-theme	Raw data exemplar
Breadth and depth of knowledge	Integration of knowledge bases	You might understand one part of it, but you have got to get the bigger picture. Physiologically, technically tactically, what are all the things that somebody needs to be able to go and stand on a podium. You can have the world’s best knowledge of physiology or anything else, but if you do not understand what it means relative to your athlete and what they need to stand on the Olympic podium, it’s pointless (C1)
	Perceived priority knowledge bases	For a coach that knows what they are talking about, they absolutely have to have brilliant sport specific knowledge, they have to understand the event demands inside out and back to front (C7)
	Knowledge of the experiences needed for progression	I remember a couple of athletes going to [competition] and [coach] sent them thinking that they were going to get on the podium; they came 30th and 40^th^. It was a complete misalignment with the expectations they are being given and the sort of performance that they are capable of delivering. The coach just did not know and one of those should have been going on to get to a very high level. (C1)
Flexible and contextualized use of knowledge	Distributed knowledge	The whole [multidisciplinary] team need to be involved. We constantly are updating and sharing information…I rely on them for their input and perspective because I cannot know everything (C4)
	Knowing what you know	We do not all [coaches] need to be experts in every area, we just need to be know enough across the spectrum. That means I also need to know my limits. Then we need to use the people who are the best at stuff (C4)
	Knowing when different knowledge applies	With my more senior athletes, the one’s I’ve coached longer, I know their behavior enough to know if they come in a bit p*ssed off, if something’s happened in their life. I can then adjust the session knowing what will work and what will not work. It’s like I know where I need to take something to keep the plan on track (C7)

#### 3.2.1. Breadth and depth of knowledge

Based on the nature of the cognitive challenges faced by coaches, there was a necessity for a significant breadth and depth of knowledge for them to be able to generate appropriate solutions to problems. Given their positioning at the center of a multidisciplinary team, this required coaches to *integrate knowledge bases*, crossing disciplinary boundaries:

It all depends, everything depends on everything else. I need to be able to know physiology and load management, but it goes hand in hand with psychological, the XXX [specifics of the sport] (C6)

This was seen as a limiting factor for less experienced coaches, that they were unable to see the interconnectedness of different domains of knowledge: “less experienced coaches struggle with using different bits of knowledge. I do not see things in isolated terms of technical, tactical, physical” (C3).

Coaches also considered that in addition to this breadth of knowledge, that there were *perceived priority knowledge bases* that coaches needed to have a deeper knowledge based on their context: “there’s got to be a fundamental understanding of physiology and athletic development” (C2). These domains of knowledge were perceived subtly differently between coaches, but there was a strong perception that knowledge of the sport should be the priority: “I think technical and teaching knowledge is very important…You [coach] really need to know enough to teach skills” (C6).

A strongly held view among the coaches was a need to have *knowledge of the experiences needed for progression.* This was the perception that coaches needed to know the types of experiences that were desirable for athlete progression, set alongside an understanding of the different routes that athletes may take in their progression. This was both the case in supporting a longer-term journey for young athletes: “you have got to know what it looks and feels like throughout the ages, and stages really well. It is not just about the top end, is about what is in between” (C2). It was also the case in progressing high-performance athletes toward being the best in the world:

You need to have the experience of working with enough people, if not, you're not going to have had enough knowledge of progression. I need to almost have a view of what I'm doing now and how it fits into that bigger picture. You might have been around as part of a support team or something like that, then you do understand the end product, but you also need to have seen people develop (C1)

#### 3.2.2. Flexible and contextualized use of knowledge

The second main theme concerned the flexible and highly contextualized use of knowledge by coaches. This involved a metacognitive weighting of different factors and the use of knowledge beyond them as an individual. In all instances, coaches did not see their own knowledge as being the key to expert practice. All coaches were highly aware of the multidisciplinary support teams around them and drew on the *distributed knowledge* around them: “I need to decide on the thing that makes the difference, but I will not always know enough without using others and the knowledge around me” (C1).

The ability to seek and access distributed knowledge was supported by coaches having a metacognitive awareness of the boundaries of their own knowledge, *knowing what you know:* “I need to know 70% of what the specialist does. That also means that I need to know what I know and also what I do not. I need to know my limits” (C3). This entailed an epistemic humility and understanding of the knowledge of others:

I wouldn't sit here and profess to have an in-depth knowledge of everything that an individual [support] practitioner does…if you bring in a physiologist they would have the theoretical underpinning, but they lack an understanding of how everything contributes to one another and how that then impacts that athlete (C3)

This was further supported by a *knowledge of when different knowledge applied*, and when it did not that was central in the coaching process:

XXX [athlete] needs to XXX [be] closer to the front….it means that the chance of a break going away at the front is minimized. It also means that he needs to expend less energy… to chase to get into the main group.. To tackle the problem, I have got to draw on principles of physiology and tactics to prepare him, then compare that to what I know he needs to do to win at a particular time (C2)

This was also seen on an individual basis, relative to the wants and needs of the athlete. Where despite believing a particular course of action to be most appropriate, what the athlete thought about a plan strongly mediated effectiveness. Thus, coaches needed to understand when and how to apply their knowledge bases, relative to the unique and individual circumstances of the athlete:

I can sit there and go ‘that's where I see your next performance gain coming from’, equally I need to know what they will invest in, or if it's not something that they want to do. I need to be sure of what I know and know at what point I would defer to what I feel that athlete would invest most in (C3)

This overall presented a complex picture of interacting knowledge types, bases and metacognitive processes that influenced the use of knowledge by coaches.

### 3.3. Discussion

Till et al. (2019) suggested that to enhance a PJDM approach for coaches, there was a need to identify “what decisions coaches need to make and what knowledge they draw upon to make such decisions” (p. 8). The specific aims of this research were to: (a) establish the cognitive demands faced by expert coaches at different levels of athlete performance and how coaches manage these demands and (b) explore the coaches’ use of knowledge to tackle the demands they face. The core insights of this paper are in identifying the nature of the types of challenges faced in this population of HP sport coaches and the knowledge that may be required to address those challenges.

#### 3.3.1. Adaptive skill

The key insight generated through the ACTA protocol is the nature and extent of coaches’ need to exercise adaptive skills to address ongoing and significantly demanding challenges in their day-to-day coaching practice. These challenges were both individually and contextually mediated (Cushion et al., 2019). Although the coaching environment was less hyperdynamic than in previous uses of CTA methods in adventure sport coaching environments (*cf*. Collins and Collins, 2013), the milieu demanded a high level of adaptability (Mees et al., 2020).

In addition to the need for adaptability in day-to-day coaching practice (Saury and Durand, 1998), coaches were highly challenged by the complex interacting factors that impact athlete development (Baker et al., 2017). Universally, the participant’s environments were highly pressurized, dynamic and ever changing (Collins and Collins, 2022). This variability included input from skill/discipline specialists, day to day changes in athlete readiness and conflicting athlete needs such as lifestyle pressures. These challenges put a significant demand on coaches to be able to make sense of cognitive challenges, both updating mental models and the application of frames for each athlete (Klein et al., 2006b). The flexible execution of intentions required coaches to engage in planning and replanning to cope with changing circumstances (Klein, 2007). Therefore, there were significant demands on the capacities of coaches to make sense of incoming data and to flexibly execute a plan that suited the needs of their athletes (Ward et al., 2018; Ashford and Taylor, 2022). Thus, our empirical data is supportive of the proposal that coaches at the highest levels of performance need to engage in significant sensemaking based on the unique and individual needs of each athlete (Simon and Richards, 2022). Going further, we also suggest that adaptive skill is a fundamental prerequisite for effective coaching practice (Ward et al., 2018).

To tackle these challenges, there were clear similarities outlined by coaches reflecting on the nature of their process against the ‘Coach Planning and Reflective Framework’ (Muir et al., 2011; Till et al., 2019). For example, coaches spent significant time reflecting on practice activities by considering the validity of challenge, purpose of training, and the intensity required for adaptation. Rather than considering training activity alone, coaches were invested in optimizing learning design more holistically. Thus, while appropriate periodization of training stress was a core feature of practice (*cf*. Kiely, 2016), coaches also reflected on activities that enabled broader adaptation, such as ‘non-physical’ indoor sessions. As a result, we suggest that the label ‘learning design’ may be more appropriate to represent the range of activities used. Furthermore, participants reflected deeply on their coaching approach, how they supported athletes to understand what they were doing and why they were doing it (Abraham and Collins, 2011a). None of the coaches planned their behaviours as discreet entities; however, they paid particular attention to the affective tone of their interactions (Taylor et al., 2022). This was perceived to be a central feature of the coaching process, especially where there was a need to cultivate a sense of credibility and competence with elite athletes (Taylor et al., 2021). Methodologically at least, this insight would suggest the need for future CTA studies to deepen our understanding of coaches’ adaptive skill.

#### 3.3.2. Interpersonal dynamics

There is an abundance of literature that has considered the interpersonal dynamics of the coaching process (e.g., Jowett and Cockerill, 2003; Cronin et al., 2018). This literature is well established and supportive of the notion that the coach-athlete relationship is a core element of effective coaching (Jowett, 2017). The insights presented here support this notion. However, the use of ACTA also provide insight into the complex dynamic of a HP coaching relationship, one that requires ongoing maintenance and response to individual needs. Interpersonal dynamics were one of the most prominent cognitive challenges, highlighting the range of Macrocognitive processes required to shape and maintain appropriate working relationships. This was further challenged by the demands imposed by the variety of stakeholders around the athlete, including other coaches, athletes and multidisciplinary staff, all of whom having the capacity to influence the athlete-coach dynamic. This suggests the need to recognize individual relational dyads as part of a wider orchestration of the milieu (*cf*. Jones and Wallace, 2006; Potrac and Jones, 2009) and the distribution of leadership facilitated by the coach (Simon and Richards, 2022).

In this sense, the interpersonal dynamics of coaching required differential approaches to each individual. For example, there was a perceived need for role clarity and professional distance to ensure both coach and athlete were aware of the function of their relationship (*cf*. De Bruyckere and Kirschner, 2016). This is not to suggest an impersonal or authoritarian stance, instead, the data strongly suggests a ‘goldilocks’ approach where both coach and athlete held a shared understanding of the function of their relationship. A prominent feature of this dynamic concerned protecting perceptions of professional credibility set against the need for humility and openness (Taylor et al., 2021). This appears especially important for the most elite athletes where coaches needed to experiment to find performance advantages, while maintaining the athlete’s belief in their competence. This presents a core challenge because while coaches cannot be sure of the outcomes of their practice, athletes may perceive a coaches’ uncertainty negatively (e.g., Jowett and Cockerill, 2003).

#### 3.3.3. Pedagogic influences

Similar insights were generated into the pedagogic positionality of the coach in relation to their athletes, considering the extent to which the coaching process was driven more by the coach or athlete. This has often been seen in in the literature as the difference between coach and athlete centered coaching (Pill, 2018). While this in itself is not a novel discovery (e.g., Mosston, 1966), the key insight relates to the complexity of sensemaking required by coaches to make decisions about their approach. This sample of coaches considered a truly biopsychosocial picture, in which their pedagogic decisions were influenced by athletes’ interoceptive awareness of their stress load (*cf*. Haase et al., 2015), what might be effective from a pedagogic perspective (Cope and Cushion, 2020) and the social norms of the sport. The latter point was particularly relevant because coaches were working with highly committed athletes who were at risk of overtraining and compromising their recovery and overall well-being (Heidari and Kellmann, 2023).

Therefore, coaches weighed input from various factors, supporting the notion of a bi-directional approach, rather than a coach or athlete-driven approach (Jowett and Slade, 2021). This required different strategies within training groups and individual athletes. Another insight from the ACTA emphasized the significant importance of an intuitive sense of normality for each athlete, along with deliberate time spent getting to know them and planning and re-planning for their development. Therefore, supporting coaches to make these decisions requires extensive knowledge across various disciplines and drawing attention to the cues presented by individual athletes.

#### 3.3.4. Knowledge

Emphasizing the utility of CTA, despite extensive literature exploring coaching knowledge (e.g., Gilbert and Côté, 2013), the use of ACTA provided a number of novel findings. A particular finding was the extent to which coaches needed to draw from multiple knowledge bases, or disciplines; with this knowledge needing to be integrated and conditionally applied (Abraham et al., 2006). This extends previous ACTA research conducted with strength and conditioning coaches, demonstrating that a key characteristic of expert practice is the possession of a broad knowledge base (Downes and Collins, 2021a). Perhaps pointing to the difference in roles, this ACTA suggests the need for the coach to have a greater breadth and depth of knowledge. In addition, having the capacity to both integrate and prioritize which bodies of knowledge might be more or less appropriate in a particular circumstance to meet desired, co-created intentions (Nash and Collins, 2006).

All coaches were supported by a team of multidisciplinary specialists, including physiologists, psychologists, physiotherapists, strength and conditioning coaches, and nutritionists. Therefore, the challenge of knowledge integration extended beyond the individual coach and posed a metacognitive challenge for coaches to understand what they knew and did not know. Coaches needed to be aware of their personal knowledge boundaries and how to draw on other specialists to better suit the needs of the athlete (Cassidy et al., 2008). The cognitive challenge of integrated practice required both adaptive interpersonal skill and the ability to weight and integrate different knowledge bases (Alfano and Collins, 2021; Burns and Collins, 2023). Interestingly, supportive of this integration, coaches drew on the importance of declarative knowledge, referring to it as ‘fundamental knowledge’ that aids application and flexibility. This declarative knowledge appeared to underpin the formation and use of conditional knowledge to meet the cognitive demands of coaches’ roles (Ashford et al., 2022).

This also suggests that expertise was contextual both to the domain of practice (high performance) and, given the need for coaches to use tacit knowledge related to individual athletes, their coaching role and milieu (*cf*. Turner et al., 2012; Robinson et al., 2022). This insight suggests a move beyond representations of coaching knowledge as static entities, progressing to an understanding of the conditional use of knowledge (Stoszkowski et al., 2020).

#### 3.3.5. Curriculum knowledge

Beyond the use of bodies of knowledge, the ACTA generated a distinct insight into coaches’ use of knowledge to mentally project desirable athlete experiences over time. This insight represents a novel finding in the coaching literature. To explain this finding, we draw on the notion of curriculum knowledge from the educational domain, something not previously developed in sport coaching (Shulman, 1986). Curriculum knowledge is split into two categories: ‘lateral curriculum knowledge’ as underpinning: “teacher’s ability to relate the content of a given course or lesson to topics or issues being discussed simultaneously in other classes” (Shulman, 1986, p. 10) and ‘vertical curriculum knowledge’ as a “familiarity with the topics and issues that have been and will be taught in the same subject area during the preceding and later years in school, and the materials that embody them” (Shulman, 1986, p. 10). Previous research in sport coaching has pointed to the importance of curricula design based on sport specific knowledge and a simplification of complexity (Abraham et al., 2022). While our findings would support the importance of beginning with contextualized (age/stage) sporting demands, a prominent feature of ACTA’s were descriptions of knowledge of desirable athlete experiences and needs (*cf*. Taylor and Collins, 2020; Taylor and Collins, 2022). This in no way suggests that coaches believed that they could consistently identify those who would be able to achieve later elite performance (*cf*. Johnston and Baker, 2022), nor that they perceived a linear step-wise pathway to high performance (*cf*. Güllich et al., 2023). Instead, coaches drew on their knowledge of previous athlete development to mentally project the needs and desirable experiences of athletes to support progression.

ACTA data suggest that coaches used horizontal curriculum knowledge to understand the breadth of an athlete’s experience at a point in time and vertical curriculum knowledge to understand needs and desirable experience at a given time and to project their needs and desirable experiences through a pathway. These findings highlight the utility of the ACTA protocol, with insights of expert/novice differences suggesting that curriculum knowledge may be one of the key differentiating factors in coaching expertise. The suggestion being that novice coaches may struggle to see the bigger picture horizontally or to mentally project an athlete’s needs vertically. However, curriculum knowledge wasn’t seen in terms of declarative or conceptual knowledge alone, coaches also need experiential or tacit knowledge of the steps taken to progress to high performance (Nash and Collins, 2006).

In practice, this subtly contrasts with previous industry guidance suggesting that pedagogic knowledge may the critical differentiator between HP and TD domains (UK Sport, 2020). This finding may be a result the context of coaches sampled, all of whom had extensive and often current experience of coaching athletes at multiple levels of performance. While additional research is needed, it may be that effective TD coaching requires a significant body of curriculum knowledge. This differs from HP coaching, where desired performance is closer and poses less of a mental projection demand, but where predictions need to be more precise. Thus, for the coach of HP athletes, the bandwidth for error was smaller, requiring a more granular knowledge of sport demands and the ability to integrate these with other knowledge bases (Abraham et al., 2006).

## 4. Implications for practice: CTA tools in sport coaching

Although used across a variety of industries, the use of ACTA in sport has thus far been very limited. The insights presented in this study suggest significant opportunity for future research to better understand expertise in coaching and for coach development in practice. Consequently, we suggest a range of opportunities for CTA tools to tackle ongoing issues in coach learning and development.

Firstly, by identifying the cognitive challenges in a specific context, there is an opportunity to shape coaches’ education and development around the most challenging cognitive dimensions of their work, thereby avoiding a coach education and development process that constrains learning and maintains cultural status quos (Serpell et al., 2023). Here, by identifying the cognitive challenges in a specific context, there is the opportunity to shape the education and development of coaches around most challenging, yet integral, cognitive dimensions of their work.

The use of CTA tools in coaching has significant potential for individual and group coach development (e.g., Hoffman et al., 2014). This is something that could be pursued more holistically, as is the case in the present study, or in specific circumstances to understand approaches adopted in practice. This could be important given the significant cultural differences between levels of performance, individual environments and sports. Tools like the Critical Decision Method could also be used as a tool to understand the less observable features of coaching practice (Taylor et al., 2023). However, the extensive nature of the data captured by CTA may make it impractical for coach developers to use with every coach. Nevertheless, adaptations to the ACTA protocol may be an effective tool for coach development work, allowing coaches to efficiently profile their needs without requiring multiple observations. Overall, there is a significant opportunity for future research to better understand expertise in coaching and to tackle ongoing issues in coach learning and development.

There are, therefore, opportunities to use CTA both locally, and more generally to identify the most cognitively challenging aspects of coaching. Locally, to support groups of coaches in a particular environment to understand the types of challenges they face. More generally, CTA can be used to understand the needs of coaches across different sports and levels of athlete performance, to support judgment and decision-making skills (Klein, 1997). For example, training coaches to use CTA for their own developmental purposes (e.g., Gore et al., 2018) in line with their preferences for social learning (Stoszkowski and Collins, 2016) and thus enhance heutagogic coach learning (Stoszkowski and Collins, 2017). In addition, this also supports the notion that expertise in coaching, is conditional (*cf*. Nash et al., 2012), something rarely applied in formal coach education experiences.

Encouraging further research, the findings also suggest possibilities for the development and resourcing of sports coaching at the macro level. For a number of years, there has been growing practical interest in the distinguishing features of effective coaching for developmental athletes (e.g., Martindale et al., 2005). The findings here may provide guidance for supporting the ‘needs’ of different domains of coaching (Lyle and Cushion, 2017). Specifically, if a coach were to specialize in talent development coaching, while knowledge of the sport, person and pedagogy may be important, curriculum knowledge may be critical to effective practice. This is not to suggest that coaches of HP athletes do not need to understand the steps necessary to progress performance, just that the typical non-linearity of development presents the need for different knowledge and extensive mental projection.

For research, although recommended for a number of years (Lyle and Vergeer, 2013), CTA tools have remained underutilized, perhaps due to the timely and iterative process they demand. There is a clear opportunity for CTA tools to enhance the relative paucity of ecologically valid research in the coaching domain. Alongside other methods, including observation (Cushion et al., 2012; Taylor et al., 2023) there is a strong case to be made for the increasing use of CTA methods to develop the evidence base for sport coaching practice. We would also suggest that coaching researchers take advantage of the flexibility offered by CTA tools for the purpose of data capture in different circumstances, and over different timepoints, similar to adaptations made in other domains (e.g., Minotra and Feigh, 2017).

## 5. Limitations

Although ACTA is well established as a method for pragmatically accessing the cognition of practitioners, the limitations of this study are no different from others that have adopted retrospective methodologies to understand ‘cognition in the wild’ (Martindale et al., 2017). Furthermore, the level of analysis conducted in this study aims to capture an overall picture rather than focusing on the minutiae of the coaching process. It is important to note that we have not sought to identify cognitive challenges that are generalizable across HP coaching roles. Instead, we ask the reader to consider transferability of findings to parallel domains, both in terms of research methodology and for coach development practice.

To judge transferability, we draw the reader’s attention to the population making up the sample and their specific context (Levitt et al., 2017). All participants were expert coaches (Nash et al., 2012) with significant experience coaching developmental and elite endurance athletes (Swann et al., 2015; McAuley et al., 2022). All were sampled from a single national governing body with a significant track record of success at World, Olympic and Paralympic level. The findings may therefore be less transferable to alternate coaching domains, performance cultures, or with participants of different motivations (Collins et al., 2012b). As a means of comparison, we encourage the use of similar methods in other coaching populations.

## 6. Conclusion

Using an ACTA approach, this study provides valuable insights into the challenges faced by high-performance sport coaches ‘in the wild’. We identify ongoing ‘cognitive challenges’, including: making sense of individual context, planning for contextual priorities, managing athlete stress, knowing when to push and pull, managing the coach-athlete relationship, and orchestrating inputs to the athlete. ACTA also identified the range of cues and strategies used to navigate these challenges, along with perceived expert-novice differences.

Adaptive skill was found to be crucial in meeting the needs of individual athletes and managing relationship dynamics within the HP context. Furthermore, reflexive thematic analysis was used to explore coaches’ use of knowledge in practice. Here, coaches used a breadth of knowledge from a variety of disciplines in a flexible and highly contextualized manner underpinning various mental processes. A novel finding being coach’s use of curriculum knowledge to support the mental projection of athlete needs. As is emphasized across the literature, our findings support the position that navigating complexity with a fixed position is unlikely to be an appropriate strategy. Our findings also emphasize the importance of adapting to complexity and highlight the differential needs of coaches at different levels of athlete performance. Based on this novel use of ACTA, we make recommendations for future use of CTA tools in sport for both research and practical purposes.

## Data availability statement

The datasets presented in this article are not readily available because data is not available on request to protect participant anonymity and competitive advantage. Requests to access the datasets should be directed to jamie.taylor@dcu.ie.

## Ethics statement

The studies involving human participants were reviewed and approved by Dublin City University (REC/2022/171). The patients/participants provided their written informed consent to participate in this study.

## Author contributions

JT, MA, and MJ contributed to conception and design of the study. JT and MA conducted interviews, data analysis, and wrote sections of the manuscript. JT wrote the first draft of the manuscript. All authors contributed to manuscript revision, read, and approved the submitted version.

## Funding

The research was supported by Open access publication funded by Dublin City University.

## Conflict of interest

JT and MA were employed by Grey Matters Performance Ltd. United Kingdom. MJ was employed by the British Triathlon Federation.

## Publisher’s note

All claims expressed in this article are solely those of the authors and do not necessarily represent those of their affiliated organizations, or those of the publisher, the editors and the reviewers. Any product that may be evaluated in this article, or claim that may be made by its manufacturer, is not guaranteed or endorsed by the publisher.
